# Chemical recycling of polyolefins: a closed-loop cycle of waste to olefins

**DOI:** 10.1093/nsr/nwad207

**Published:** 2023-08-02

**Authors:** Liang Zou, Run Xu, Hui Wang, Zhiqiang Wang, Yuhan Sun, Mingfeng Li

**Affiliations:** Sinopec Research Institute of Petroleum Processing Co., Ltd, Beijing 100083, China; Sinopec Research Institute of Petroleum Processing Co., Ltd, Beijing 100083, China; Shanghai Advanced Research Institute, Chinese Academy of Sciences, Shanghai 201210, China; 2060 Research Institute, ShanghaiTech University, Shanghai 201210, China; Sinopec Research Institute of Petroleum Processing Co., Ltd, Beijing 100083, China; Shanghai Advanced Research Institute, Chinese Academy of Sciences, Shanghai 201210, China; 2060 Research Institute, ShanghaiTech University, Shanghai 201210, China; Sinopec Research Institute of Petroleum Processing Co., Ltd, Beijing 100083, China

**Keywords:** polyolefins, pretreatment, chemical recovery, product refining, life cycle assessment

## Abstract

The unsuitable disposal of plastic wastes has caused serious environmental pollution, and finding a green manner to address this problem has aroused wide concern. Plastic wastes, especially polyolefin wastes, are rich in carbon and hydrogen, and chemical recycling shows distinct advantages in their conversion into olefins and realizes a closed-loop cycling of plastic wastes. Plastic wastes should be labeled before disposal. The necessity for, and methods of, pretreatment are introduced in this paper and the whole recycling process of polyolefin wastes is also summarized. As the core technology pyrolysis, including thermal, catalytic and solvolysis processes, is introduced in detail due to its potential for future development. We also briefly describe the feasible strategies of pyrolytic oil refining and life cycle assessment of the chemical recycling process. In addition, suggestions and perspectives concerning the industrial improvement of polyolefin chemical recycling are proposed.

## Teaser

This paper gives an overview of the whole chemical recycling process of polyolefin wastes, including the pretreatment of feedstocks, chemical recovery, product refining to olefins, and LCA analysis of chemical recycling.

## INTRODUCTION

Compared with steel, wood and cement, plastic exhibits excellent advantages, such as durability, light weight, strong plasticity, energy efficiency and other properties which enable it to be widely used in construction, transportation, electrical and electronic, agriculture, packaging, medical and other industries. By 2022, the total global plastic output had reached 367 million tons [1], and 50% of plastic products are estimated to end up in the waste stream after being used only once or entering the circulation domain for a short period of time [2]. The service life of several plastic products, such as packaging, bags, and utensils, is less than one month [3]. Household plastic waste streams mainly include five types of plastics, namely, polyethylene (PE), polypropylene (PP), polyvinyl chloride (PVC), polystyrene (PS) and polyethylene-terephthalate (PET), which account for 74% of all plastic wastes [4]. Given their biological and chemical inertness, the degradation period of these plastic products in the natural environment is long, and can be further prolonged due to the addition of antioxidants and stabilizers during production. As a result, plastic wastes, especially packaging bags, accumulate in the natural environment or landfills, which causes severe environmental pollution, such as air, groundwater and marine pollution [5]. Although biodegradable polymers can be one of the solutions to the problem of plastic wastes, only 2.6 million tons of biodegradable polymers are estimated to be produced by 2023 (less than 1% of the demand for plastic) [6]. Furthermore, their production costs, mechanical properties, and chemical durability are incomparable to those of petroleum-based plastics. In addition, the degradation rate of biodegradable polymers in the natural environment remains poor. Therefore, the recovery and high-value utilization of plastic waste is the most important means to transform wastes into valuable materials and solve the problem of ecological and environmental pollution [7].

Given the increased amount of plastic waste, several recycling/recovery methods, including energy recovery, mechanical recycling and chemical recycling, have been proposed. Energy recovery by incineration is a suitable solution taking advantage of the high energy content of plastics. However, this process leads to the possible emission of toxic compounds and a large amount of carbon dioxide. Compared with energy recovery, mechanical recycling and chemical recycling of plastic wastes have attracted considerable attention in the context of carbon emission reduction. Mechanical recycling is widely used in the treatment of single-component plastic wastes, but it is difficult to use in the recovery of plastics with high performance, multiple functions and complicated components. Chemical recycling can depolymerize plastic wastes into their monomers, which can then be used as feedstocks to produce virgin plastics. However, chemical recycling of plastic wastes comes with a number of challenges, such as how to collect and sort low-value plastic wastes, difficulty of obtaining pyrolysis products with properties that meet refinery requirements and the lack of a suitable business model. However, from the perspective of sustainable development, the advantage of chemical recycling will promote its rapid development in the coming decades.

**Figure 1.**
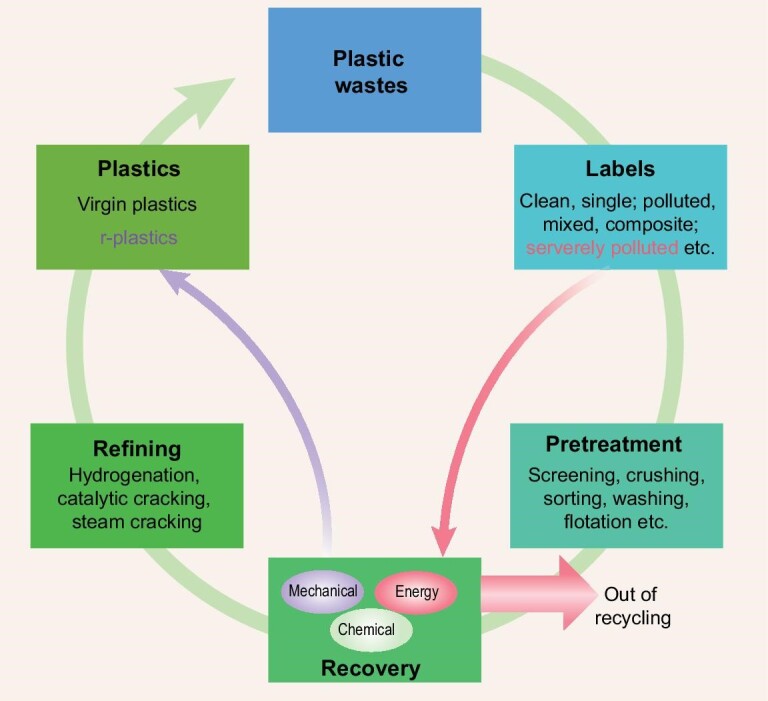
Recycling strategies of plastic wastes.

Plastic waste management aims at using a hierarchical approach. As shown in Fig. 1, plastic wastes must be labeled as clean, polluted, single, mixed, composite, etc., followed by appropriate pretreatments like sorting, compressing, crushing and pelletizing to produce raw materials. Targeted recycling schemes should be selected for different labeled plastic wastes. Severely polluted and mixed plastic wastes with extremely complex compositions are suitable for energy recovery. Clean and single plastic wastes are preferred in the production of flakes or granules through mechanical recycling. Chemical recycling is an effective method for the closed-loop cycling of slightly polluted mixed or composite plastic wastes.

In theory, chemical recycling approaches can be used in the infinite recycling of polymers into monomers. However, notable technical and economic challenges exist for the fully circular economy of commodity plastics, in particular polyolefins. In terms of thermodynamic factors, saturated PE and PP are generated by the addition of polymerization reactions; however, numerous polymerization reactions require an extremely large Gibbs free-energy change for the complete reconversion of polymers to monomers [7]. As a result, a number of undesirable side reactions with low activation energy occur. Thus, the recovery of monomers from waste polyolefins through pyrolysis produces complex mixtures that require further processing.

In the past several decades, reviews have summarized the chemical recovery of polyolefin wastes, and most researchers have used pure resin as feedstocks, ignoring the source and composition complexity of actual polyolefin waste streams. This review aims to provide insights into the whole process of chemical recycling, including pretreatment, chemical recovery, and product refining to olefins and highlights the main progress in the chemical recovery of polyolefin wastes. The green properties of the chemical recycling of polyolefin wastes are explained through LCA analysis, and its future development direction is proposed. This review can help researchers determine the direction for their research, and practitioners to select appropriate technical solutions in order to establish the chemical recycling industry chain of polyolefin wastes.

## PRETREATMENT OF POLYOLEFIN WASTES

**Figure 2.**
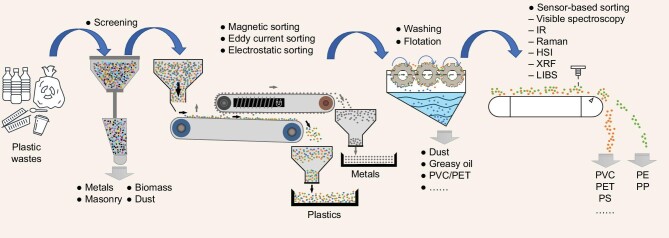
Chemical recycling pretreatment scheme.

The pretreatment of polyolefin wastes is an essential step of chemical recycling, although it brings additional costs. On the one hand, this process is advantageous in improving the stability of unit operation; on the other hand, it enhances the quality of pyrolysis products and maintains the economic feasibility of the related processing plants, such as those plants used for pyrolysis and refining. The primary pretreatment of polyolefin wastes includes the removal of extraneous materials and nonpolyolefin plastics (Fig. 2).

The removal of extraneous materials involves screening, sorting, washing and flotation, and the size of polyolefin wastes must be uniform in order to efficiently optimize removal [8]. Metals can be removed by magnetic, electrostatic and eddy current separation techniques. The washing process includes coarse/rough and fine washing. Most of the sediment and water-soluble impurities adhering to polyolefin wastes can be removed by coarse washing, and alkali or acid washings are typical methods of fine washing that improves the cleaning quality. However, 2–3 m^3^ water is required to clean 1 t of polyolefin waste in the latest generation of wash plants, which brings about additional costs and a substantial quantity of wastewater [9]. Table 1 shows the technical principles and characteristics of these methods [10–13].

**Table 1.** Brief introduction of the main sorting methods.

Method	Technical principles	Characteristics	Ref.
Drum screen	The input material is loosened and redistributed by the rotary motion, which exposes enclosed particles and separates the undersize particles from fine to coarse.	Low separation efficiency; impurities can be coated by two-dimensional (2D) films; adhesive wet impurities are difficult to remove; screening efficiency of 70%–90%; approximately 15% degree of filling in drum screen.	[10]
Magnetic sorting	Change the motion state of feedstocks by the difference in magnetic induction forces in the magnetic field.	Can only remove ferromagnetic metals in feedstocks. The most efficient separation for grain class (0.32–0.1 mm).	[11]
Electrostatic sorting	Based on the electrical properties of each component in a high-voltage electric field.	Used for sorting conductive, semiconductive and nonconductive materials; can also be used to sort different types of plastics. The diameter of feedstocks is 0.04–1 mm. Requires more than 10 000 V static voltage and dry materials.	[12]
Eddy current sorting	Conductors can generate induced currents in high-frequency alternating magnetic field, generate a magnetic field in the opposite direction of the original one, and finally form a repulsive force.	Used for sorting nonferrous metals with particle size > 2 mm; iron must be removed first; high-strength permanent magnet materials effectively save electricity.	Open data
Flotation	Utilizes the difference in floatability of fine-grained materials to achieve separation.	Suitable for sorting plastic mixtures with little or no density contrast of components; the product purity can reach over 90%, and the recovery rate is 80%–99%. Suitable range of particle size depends on the flotation device. Inclusion of additional chemicals poses a risk of water pollution.	[13]
Wind separation	Based on the difference in suspension speed between materials and impurities.	Always combined with cyclone separator; low sorting efficiency of 40%–70%. Susceptible to moisture content, bag breakage rate, and entanglement conditions and uniformity; needs a uniform particle size.	Open data
Visible spectroscopy	Based on visible spectra and performed in the wavelength range 400–700 nm or adopted in digital imaging.	Low separation efficiency; always used for color separation of clean monomaterial steam.	[17]
IR spectroscopy	Based on the difference in characteristic peaks of different functional groups in near-IR (NIR) (780–1100 nm) and middle IR (MIR) (1100–2526 nm) bands.	High detection efficiency, online nondestructive testing and harmless to humans; MIR is unaffected by black plastics. Susceptible to environmental interference.	[18,19]
HSI	Based on image data technology of many narrow bands; it combines imaging technology with spectral technology to detect 2D geometric space and 1D spectral information of targets and obtain continuous and narrow band image data with a high spectral resolution.	Rich spectral information, high spatial and spectral resolution, numerous bands and abundant information; reduction in analysis time and objective analysis; high dimensionality of HSI data.	[20,21]
Raman spectroscopy	The frequency of the interaction between photons and molecular changes and the light signal scattered from the sample surface are collected to obtain relevant information on the detected molecules.	High detection efficiency; overcomes the shortcomings of NIR. Background fluorescence of Raman often overshadows several characteristic peaks.	[22]
X-ray fluorescence	Identification of plastic species based on X-ray spectrum of characteristic atoms in plastic.	High sorting efficiency for halogen-containing plastics. Easily affected by mutual element interference and superimposed peaks.	[23]
LIBS	Plasma is formed on the sample surface by focusing the ultrashort pulse laser, and the plasma emission spectrum is analyzed to determine the material composition and content of the sample.	Sensitive to specific chemical bonds and has high accuracy and wide range of identifiability. Easily affected by similar element composition and additives in plastic.	[19]

Nonpolyolefin polymers, such as PVC, PET and acrylonitrile butadiene styrene, should be removed to reduce their effect on pyrolysis. This process usually includes flotation and sensor-based separation. Flotation is a simple and effective method to separate different kinds of plastics. The quality of separation is related to reagent selection and varies with the mixture type; it depends not only on the hydrophobicity of the plastic but also on the size, density and shape of particles [14]. Plastic particles with small size, lamellar shape and low density show desirable floatability [15,16]. Sensor-based sorting is an automated sorting method based on the differences in spectral properties of plastics, including visible spectroscopy, infrared (IR) spectroscopy, hyperspectral imaging (HSI), Raman spectroscopy, X-ray fluorescence and laser-induced breakdown spectroscopy (LIBS). Table 1 shows the technical principles and characteristics of these methods [17–23]. The interference of noise, background factors and extraneous materials can affect the quality of collected data and thus the sorting accuracy for actual nonpolyolefin wastes. In terms of sorting practices, multiple technologies are usually needed to improve the efficiency of separation.

A number of laboratory studies have focused on the sorting technology of plastic wastes; thus, the detailed content will not be elaborated further in this study. The chemical recycling technology of plastic wastes, which is currently in the pilot or industrial demonstration stage, requires high-quality feedstocks. Thus, it is necessary to pretreat the feedstocks to meet the feeding requirements. The Hydro-PRT^®^ developed by Mura requires a polyolefin content higher than 80% and PVC, PET, moisture, and contaminant (paper, metals and organic waste) contents lower than 0.5%, 5%, 5% and 5%, respectively.

The energy consumption and cost of pretreatment technology, which depends on the properties of feedstocks, are relatively limited. Using the plastic waste excavated from landfills as an example, the comprehensive cost of sorting plastic waste in order to get impurity and moisture content below 5% is approximately 600–800 RMB/t. The price of sorted miscellaneous films can reach 1000–1500 RMB/t. In addition, given the limitations on word count requirements in this special issue, only feasible disposal methods are provided for reference.

## CHEMICAL RECOVERY PROCESS

Typical chemical recovery methods of plastic wastes include pyrolysis and gasification. Plastic pyrolysis involves the heating of raw material in a pyrolysis reactor to break the C–C and C–H bonds in the absence of oxygen and its conversion into small-molecule products dominated by hydrocarbons [24]. As plastic wastes are derived from the petrochemical industry, their conversion back into petroleum fractions or other petrochemical products has a high atomic efficiency. Gasification of waste polyolefins leads to the production of a stream made up of mainly H_2_ and CO. In general, the production of liquid hydrocarbons or syngas from polyolefin wastes by adjusting the reaction temperature is technically feasible, and both products can be further used to prepare olefins, which contributes to the circular economy by returning plastic wastes back into the cycle loop.

### Thermal pyrolysis

**Figure 3.**
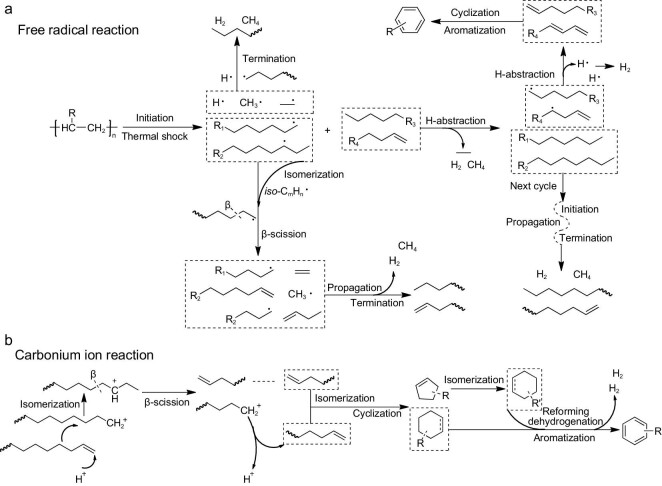
Proposed reaction mechanisms of polyolefins during thermal pyrolysis: (a) free radical reaction and (b) carbonium ion reaction. Reproduced from ref. [25].

Thermal pyrolysis of polyolefins mainly follows the free-radical reaction mechanism, which involves three stages: chain initiation, propagation and termination [25]. First, the initial thermal shock causes the fracture of any C–C bonds along the PE chain, which results in the formation of primary free radicals and reduction of the molecular weight of polymers (Fig. 3a) [25]. In the second stage, new radicals are generated due to H-abstraction reactions between primary free radicals and hydrocarbons and are further converted into olefins through the decomposition of C–H bonds. The obtained olefins cause the formation of aromatics through cyclization and aromatization reactions. In addition, several free radicals that undergo isomerization reactions stabilize and may break down into a large amount of liquid olefins and new radicals through further β-scission. In the termination stage, interactions occur by disproportionation or recombination of existing radicals, which results in the formation of H_2_, CH_4_, alkanes and short-chain alkenes.

Numerous studies have reported the pyrolysis characteristics of polyolefins, and the reviews of product distributions are summarized in Table 2 [26–39]. The factors affecting the pyrolysis of plastic wastes mainly include reactor type, feedstock, reaction temperature, residence time and pressure. The complex behavior of polyolefin wastes with low thermal conductivity, sticky nature, and low softening and melting temperatures during pyrolysis brings a great challenge for the development of suitable reactors. In general, two crucial principles, such as improving the heat transfer rates and lowering the residence time, are preferred in the design of pyrolysis reactors, which help in the production of more liquid oil and inhibit the secondary reactions that form more char or gaseous products. Fixed bed reactors have a low heat transfer rate and long residue time and noncontinuous operations limit their widespread application [40]. Meanwhile, these reactors cannot maintain uniform temperature profiles, especially in the pyrolysis of large volumes of polyolefin wastes. Fluidized bed reactors allow strong heat and mass transfer, and they are suitable for continuous isothermal pyrolysis with relatively low residence time and operating cost [41]. Park *et al.* [42,43] designed a fluidized bed reactor to prepare aromatic hydrocarbons from PE and PP and observed that the aromatic selectivity in pyrolysis oil was as high as 92%. Salaudeen *et al.* [44] developed an internally circulating fluidized bed reactor to reduce the residence time of plastic wastes and improve the selectivity of olefins and demonstrated its validity. However, several disadvantages, such as particle size limitations and attrition of bed material during operation, have been recorded. Compared with fluidized bed reactors, conical spouted bed reactors have lower attrition and bed segregation due to the tight interaction between phases, and the substantially increased collision rate between particles decreases the agglomeration. Furthermore, this type of reactor can adapt to applications with a wide particle size distribution, large particles and different particle densities [45]. Meanwhile, the residence time of feedstocks in conical spouted bed reactors is reduced to approximately 20 ms, which can effectively avoid the occurrence of secondary reactions and reduce the formation of coke. Orozco *et al.* [46] confirmed that these reactors are suitable for the pyrolysis of plastic wastes given their hydrodynamic behavior and versatility. However, the complex construction process and a variety of pumps in the system result in high investment costs. Screw kiln reactors can operate in continuous mode and control the residence time of feedstocks by changing the rotation speed of the screw. During pyrolysis, an inert gas is fed into the reactor to ensure the absence of oxygen in the system, which contributes to the transport of pyrolysis steam [46]. In addition, solid carriers can be placed in the reactor to further improve the efficiency of heat and mass transfer, which results in the closer interaction between feedstocks. However, screw kiln reactors have the risk of plugging, possible heat transfer difficulties at large scales and poor mixing in the radial direction [47]. Compared with the above conventional reactors, microwave reactors have numerous advantages. Microwave energy can be applied directly to feedstocks through molecular interactions with the electromagnetic field, which increases the heating rate of the pyrolysis process. The low dielectric constant of plastics can reduce the efficiency of microwave heating; thus, a microwave-absorbent material should be mixed with plastic wastes during pyrolysis. Zhou *et al.* [48] designed a 200 kg/day laboratory-scale continuous microwave pyrolysis reactor, used high-temperature SiC balls to heat HDPE chips and confirmed that the reactor can effectively improve heat transfer efficiency and achieve fast pyrolysis. The different heating efficiencies of each material limits the industrial application of this technology. In addition, molten salt reactors [38], induced plasma reactors [39] and screw extruder reactors used for polyolefin pyrolysis have been developed. Screw extruder reactors are normally used for densification, viscosity reduction and transportation of polyolefin wastes. A single-screw extruder is the most mature type of extruder at the moment, and it is widely used in polymer extrusion processes with the advantages of high speed, high performance, stable extrusion and low equipment cost. The screws of a twin-screw extruder (TSE) typically engage with each other and rotate in the same or opposite directions. By comparison, the corotating TSE offers higher capacity and better thermal management and cleaning capabilities, and Sinopec has applied it as visbreaking equipment for the chemical recovery of plastic wastes. The plastic wastes enter the extruder through a feeder and are rapidly heated to a melt at a temperature of 350^o^C. These plastics are further heated to 500^o^C after entering the coking reactor, and the polymer chain is split into smaller monomers in an oxygen-free environment. The combination process of visbreaking and coking developed by Sinopec has improved the yield of pyrolysis oil to a certain extent.

**Table 2.** Product distributions in the thermal pyrolysis of plastics.

					Yield of product (%)				
Feedstock	Reactor	T (^o^C)	Residence time or Feed rate	Pressure	Oil	Wax	Gas	Residue	Ref.
HDPE	Fixed bed reactor	550	—	—	70 (oil + wax)	—	22	7	[26]
PP	Fixed bed reactor	525	—	Atmospheric	76.4	4.2	10.1	2.6	[27]
PP	Fixed bed reactor	525	—	Vacuum	70.8	20.5	5.1	2.4	[27]
HDPE:LDPE:PP = 25:35:40	Fixed bed reactor	500	60 min	—	30.7 (oil + wax)	—	67.9	1.4	[28]
PE	Fluidized bed reactor	500–600	12.4–20.4 s	—	81.2–28.5	—	8.2–56.8	10.6–14.7	[29]
HDPE	Fluidized bed reactor	640–850	3–4 g·min^−1^	—	68.5–16.2	—	33.5–89.1	—	[30]
PP	Fluidized bed reactor	510	14 g·min^−1^	—	10.5	86.6	2.7	0.17	[31]
PE	Fluidized bed reactor	510	16 g·min^−1^	—	30.1	63.1	6.5	0.26	[31]
Mixed municipal plastic wastes	Screw kiln reactor	500	5 g·min^−1^	—	5.5	93.2	1.3	—	[32]
PP	Screw kiln reactor	500–700	10 min	—	50–20	—	6–80	—	[33]
LDPE	Screw kiln reactor	500–550	1.5 g·min^−1^	—	84.6–79.3	14.4–13.6	1.0–7.1	—	[34]
HDPE	Conical spouted bed reactor	500–700	1 g·min^−1^	—	31.4–49.3	67–11.3	1.6–39.4	—	[35]
PE	Conical spouted bed reactor	450–600	1 g·min^−1^	—	20–49 (oil + gas)	80–51	—	—	[36]
PP	Conical spouted bed reactor	450–600	1 g·min^−1^	—	8–50 (oil + gas)	92–50	—	—	[36]
HDPE	Microwave reactor	500	—	—	74.7 (oil + wax)	—	13.3	12.0	[37]
PP	Microwave reactor	600	—	—	83.9 (oil + wax)	—	15.7	0.4	[37]
HDPE	Molten salt pyrolysis reactor	408–423	—	—	94–92 (oil + wax)	—	6∼8	—	[38]
PP	Molten salt pyrolysis reactor	362–417	—	—	98–96 (oil + wax)	—	2∼3	—	[38]
LDPE	Induced plasma reactor	550	30 min	—	56.9	—	37.8	5.3	[39]
LDPE	Screw extruder reactor + Coking reactor	500	30 min	—	93.8 (oil)	—	5.1	1.1	Sinopec

The pyrolysis of polyolefin wastes is a typical endothermic reaction, and temperature is one of the most significant operating parameters affecting the distribution and quality of products. The appropriate increase in temperature favors the production of liquid oil and gases, while an excessively high temperature will lead to further cleavage of macromolecular pyrolysis products to small-molecular gases. The aliphatic content in the oil sharply decreases, and those of benzene, toluene and xylene (BTX) aromatics increase at high temperatures because of the enhancement of Diels–Alder and dehydrogenation reactions [49]. During the pyrolysis of polyolefin wastes, the target product, such as wax, light olefin or BTX, can be obtained by reactor design and adjusting the operating parameters [50]. The yield of the target product waxes increases under temperatures less than 500°C and short residence times of volatiles [40]. The production of light olefins requires higher reaction temperatures in the range of 800°C to 950°C. During processing, the target temperature should be reached rapidly to facilitate the deep cracking of polyolefin wastes, and the residence time of volatiles should be kept to a minimum in order to inhibit other side reactions. BTX are usually formed by the synergistic effect of the Diels–Alder reaction of small molecular olefins in primary pyrolysis products and the dehydrogenation reaction at temperatures above 700°C [50]. However, the aromatization of olefins and condensation of monocyclic aromatics into polycyclic aromatics (PAHs) are sensitive to the residence time, which results in the production of low-yield BTX by direct thermal pyrolysis; in addition, controlling the generation of PAHs in the process is difficult [51].

Pressure and residence time are also significant operating parameters for the pyrolysis of polyolefin wastes. Pressurized conditions are conducive to aromatization and oligomerization reactions, which promote the formation of aromatic hydrocarbons [51]. Nevertheless, these reactions decrease the selectivity of light aromatics and facilitate the generation of heavy aromatics as coke precursors at elevated pressures [45]. Residence time shows similar effects on the pyrolysis of polyolefin wastes under reaction temperatures. Long residence times promote the conversion of polyolefin wastes into light hydrocarbons and noncondensable gases. It also increases the chance of secondary reactions, such as further cracking of the primary products and isomerization, cyclization and aromatization reactions [28].

To a certain extent, the quality of pyrolysis oil can be improved by adjusting the reaction parameters. However, the pyrolysis of polyolefin wastes follows the rule of random chain breaking, which results in a complex and wide range of pyrolysis oils (C_5_–C_60_). The liquid products need to be further upgraded before replacing traditional fuels in chemical production. Thus, finding a mechanism on how to effectively increase the content of valuable components in pyrolysis oil is a key scientific problem that must be solved for the direct preparation of high-quality liquid fuel from waste polyolefins.

### Catalytic pyrolysis

**Figure 4.**
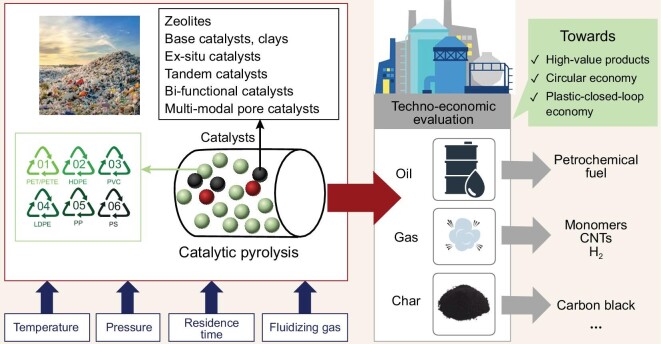
Catalytic pyrolysis of polyolefin wastes to high-value products. Reproduced from ref. [45].

The catalytic pyrolysis of polyolefin wastes is a thermal cracking process that selectively breaks polymer molecular chains, in which the reaction process and product distribution are tunable with different catalysts (Fig. 4) [45]. With the addition of catalysts, the cracking of long-chain substrates is accelerated at low reaction temperatures and short reaction times [52], which result in concentrated product distribution and energy savings. At present, catalysts with acid sites and pores [53], such as zeolites (ZSM-5, HY, beta, HUSY, etc.), activated carbon, fluid catalytic cracking (FCC) equilibrium catalysts and metal oxides, have been used to increase the yield of targeted products and change the mechanism of catalytic pyrolysis (Table 3) [54–60]. Specifically, the acidity strength of catalysts contributes to the cracking and aromatization of polymers, which determines the reaction process and yield of targeted products. To a certain extent, the porous structure of catalysts affects the molecular weight of the corresponding intermediates, and a large pore size results in long-chain products [61].

**Table 3.** Catalytic performance of different plastics under different reaction conditions.

				Yield of product (%)				
Feedstock	Catalyst	Reactor	T (°C)	Alkenes	Alkanes	Gasolines	Aromatics	Ref.
PE	HZSM-5	Fixed bed (*in-situ*)	600	28	34	—	27	[54]
PE	HZSM-5	Fixed bed (*ex-situ*)	600	80	7	—	11	[54]
HDPE	Hβ	Batch reactor	480	65	30	83	5	[55]
HDPE	HY	Batch reactor	480	30	45	60	25	[55]
HDPE	HZSM-5	Batch reactor	480	50	25	66	25	[55]
HDPE	Al-MCM-41	Fixed bed	450	51	37	56	4	[56]
PE	HY	Fixed bed	400	31.7	34.9	—	2	[57]
PE	HY	Fixed bed	500	34.2	30.8	—	2.3	[57]
HDPE	FCC equilibrium catalyst	Batch reactor	350	52.6	34.4	47.7	13.0	[58]
LDPE	Activated carbons	Fixed bed	500	—	71.8	—	28.2	[59]
LDPE	Al_2_O_3_ + M-clay	Batch reactor	30–600	17	60	70	14	[60]

**Figure 5.**
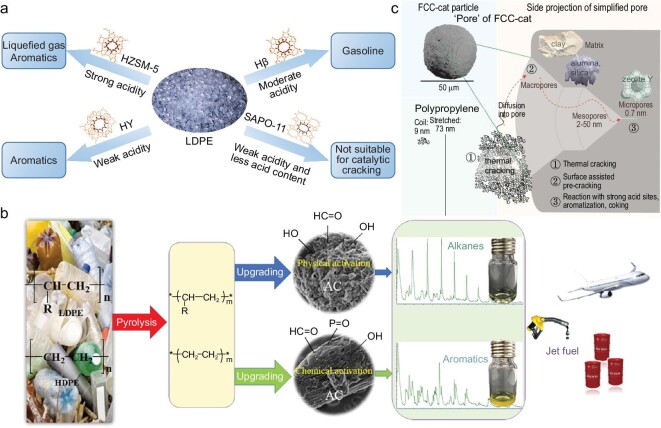
(a) Typical zeolites used for LDPE catalytic pyrolysis. Reproduced from ref. [55]; (b) the proposed reaction route of converting polyolefin wastes into transportation jet fuel over activated carbon. Reproduced from ref. [59]; (c) structure illustration of FCC catalysts. Reproduced from ref. [63].

Typically, zeolites play a substantial role in the catalytic pyrolysis of polyolefin wastes (Fig. 5a) [55]. HZSM-5 zeolite has been applied in the conversion of PE into liquid fuels [54]. The yields of alkene and alkane reached 80% and 7%, respectively, during *ex-situ* catalytic pyrolysis, and 28% alkenes and 34% alkanes were produced during *in situ* catalytic pyrolysis. The aromatic yield was 27% during the *in situ* catalytic pyrolysis of PE, and it decreased to 11% when PE during *ex-situ* pyrolization. In the catalytic pyrolysis of PE using HZSM-5, the olefins generated from PE decomposition can be aromatized, and the aromatization reactions released free hydrogen atoms [54]. The hydrogen atoms contribute to the thermal decomposition of PE and the saturation of olefins into alkanes. *In situ* catalytic pyrolysis can promote the aromatization of olefins inside the catalyst pores and the alkane yield was higher because of more hydrogen atoms for the saturation reactions. During *ex-situ* catalytic pyrolysis, catalytic cracking of PE was the predominant reaction, resulting in high olefin yields in liquids. Wang *et al.* [55] observed that HZSM-5/HY/Hβ catalysts showed high alkane and alkene selectivity but low aromatic selectivity in liquids during the catalytic pyrolysis of HDPE at 480°C. Aguado *et al.* [56] reported a similar conclusion during the catalytic pyrolysis of LDPE with Al-MCM-41.

Activated carbons, which possess a large surface area and porosity, have excellent application prospects in the catalytic pyrolysis of polyolefins [62]. Zhang *et al.* [59] employed different activated carbons in the deconstruction of LDPE and proved that catalysts with weak and relevant strong acidity favored jet fuel-range alkanes and aromatics, respectively (Fig. 5b). In addition, the selectivity of alkanes and aromatics reached 71.8% and 28.2%, respectively, the aromatization of alkanes was enhanced and more H_2_ was released at higher temperatures.

FCC catalysts are composed of active components (generally zeolites, such as Y and ZSM-5 zeolites) and the matrix (clay, alumina, silica, etc.) (Fig. 5c) [63]. Given the high density of Brønsted acids, the catalyst is active for cracking and aromatization. Mesopores in the FCC catalyst allow large molecules to access the inside of the catalyst for precracking and provide Lewis acid sites for aromatization. Therefore, it has considerable potential for the conversion of plastic wastes into high-value-added products, such as diesel oil and gasoline [63].

Considering the different mechanisms of acid and base sites, metal oxides or metal salts exhibit high activity in the catalytic pyrolysis of polyolefins; CuCO_3_ contributes 94% yield of liquid hydrocarbons [64]. Compared with a single catalyst, a narrower product distribution from the deconstruction of LDPE was obtained under the relay catalysis mode using Al_2_O_3_ catalysts followed by ZSM-5 at 550°C. During the process, polymers penetrated the large pores of the Al_2_O_3_ catalyst for cracking into lighter hydrocarbons, which can enter the micropores of ZSM-5 for further reformation, which contributed to C_5_–C_12_ alkanes/olefins with a selectivity up to 77% [60]. The synergy of the two catalysts narrowed the production distribution. Recent studies have reported the strategy of converting PE into high-value products under mild conditions. Tennakoon *et al.* [65] designed a catalyst with a mesoporous shell/active site/core (mSiO_2_/Pt/SiO_2_) structure, and the active site of platinum nanoparticles was located at the bottom of the mesopore in the silica shell. The long chain of HDPE moved in the hole, and the active site can catalyze its hydrogenolysis reaction. The small molecular products were desorbed from the end of the long chain and escaped from the hole. At 300°C, 24 h and 1.38 MPa H_2_, HDPE was converted into small molecular alkane segments with narrow chain lengths, and the products can be used for the preparation of diesel and lubricating oil. Duan *et al.* [66] reported the low-temperature conversion of PE into olefins. They mixed the PE feed with rationally designed ZSM-5 zeolite nanosheets at 280°C with flowing hydrogen as a carrier gas and obtained a yield of up to 74.6% light hydrocarbons (C_1_–C_7_) in which 83.9% of these products were C_3_–C_6_ olefins with almost undetectable coke formation. In addition, Li *et al.* [67] designed a tandem catalytic conversion of LDPE into naphtha with beta zeolite and silicalite–1 encapsulated Pt nanoparticles as catalysts, which provided an 89.5% yield of naphtha and a 96.8% selectivity of C_5_–C_9_ hydrocarbons under mild conditions. These new strategies can realize the high value-added utilization of polyolefin wastes, but the reaction time must be extended to improve the yield of the target product.

In summary, the acidity, pore size and active metal of catalysts have significant effects on the yield and composition of catalytic pyrolysis oil from plastic wastes. Catalytic pyrolysis involves a free-radical mechanism and carbocation reaction mechanism. During the *ex-situ* reaction, plastic is first thermally cracked in a single-pyrolysis reactor, and the vapor follows the free-radical mechanism. Random scission occurs to generate free radicals along the carbon chains, and a molecule with an unsaturated end and another with a terminal free radical are formed with further cleavage. Through H-abstraction reactions, straight-chain dienes, alkenes and alkanes are obtained from the radical fragments [68]. Using solid acid catalysts, the *in-situ* reaction mechanism of the pyrolysis of polyolefins follows a carbonium ion mechanism (Fig. 3b) [25]. The cracking of polymer chains begins with protonation of Brønsted acid sites or hydride abstract on the surface Lewis acid sites to form carbocationic intermediates, followed by β-scission and skeletal rearrangement to form short-chain hydrocarbons and more isomers. With the catalysis of solid acid, cyclization, aromatization and alkylation can occur to form aromatics and significant amounts of H_2_.

From the results of the above research, the selection of catalysts for the upcycling of plastic wastes plays a critical role in catalytic pyrolysis. Catalysts drive isomerization, oligomerization cracking, and hydrogen transfer and have a large influence on product distribution. The conversion efficiency of feedstocks and the selectivity of the target product are mostly determined by the acid strength and porosity of catalysts. Microporous zeolites (HZSM-5, HY, Hβ, etc.) can considerably increase the yield of gaseous products during polyolefin pyrolysis, which results in a low yield of liquid products. Mesoporous zeolites (MCM-41, SAHA, etc.) have a higher activity in the selective production of liquid fuels than microporous catalysts, but they show lower hydrothermal stability, mechanical stability and acidity. Compared with zeolite and FCC catalysts, activated carbons have the lowest acidity but have the advantages of a simple preparation process, low cost and high thermal stability. In addition, to improve the efficiency of the catalytic pyrolysis process, most methods for modification of catalysts have been carried out, such as acid leaching and thermal and wet impregnation [69]. Furthermore, the synergy of different catalysts can tune product distribution through a combination of high porosity, high acidity, and hydrogenation properties to prevent deactivation and increase catalyst reuse.

Although the addition of catalysts shows great advantages over thermal pyrolysis, the pyrolysis oil obtained from catalytic pyrolysis of waste polyolefins is still a complex hydrocarbon mixture, and its carbon numbers range from C_6_ to above C_25_. Thus, pyrolysis oil needs further upgrading steps, such as catalytic cracking and hydrogenation, before they can be used as fuels. In addition, issues, such as how to prevent catalyst deactivation due to carbon deposition and the ability to recycle catalysts in order to reduce costs, still need further research.

### Solvolysis

Catalytic pyrolysis of polyolefin wastes can considerably improve the selectivity of target materials, which provides the possibility for further application of liquid products. However, given the poor thermal conductivity and high melting viscosity of plastics, the catalyst is prone to coke deposition and deactivation during pyrolysis. In general, compared with solvent-free pyrolysis, PE depolymerization can be promoted substantially in the presence of solvents because of the improvement in mass transfer and heat transfer rates. Meanwhile, the solvent effect may change the distribution of pyrolysis products by changing the thermal degradation mechanism. The thermal degradation of HDPE in the presence of hydrogen-donating solvents follows the free radical mechanism, as in the case of cracking in the absence of solvents. The solvent-free pyrolysis of PE contributes to the production of alkanes, and the yield of olefins is high in solvent pyrolysis. The radicals generated from solvents can follow two pathways during pyrolysis. One of the pathways is hydrogen atom abstraction from the polymer chains by solvent radicals, which leads to secondary radicals and regeneration of the corresponding solvent molecules. This effect, in turn, favors HDPE chain β-scissions and thus an increase in HDPE conversion. The other pathway is the dehydrogenation reaction of solvents [70]. Serrano's team studied the pyrolysis characteristics of HDPE in hydrogen donor solvents, such as tetrahydronaphthalene, decalin and phenol, under noncatalytic conditions and observed that the presence of solvents promoted the hydrogen transfer reaction from solvents to HDPE radicals and from molecular chains to solvent radicals, which aided in the conversion of HDPE and production of α-olefins [70–72]. Ellis *et al.* [73] and Jia *et al.* [74] reported that PE was degraded into transportation fuels and waxes through a tandem catalytic cross-alkane metathesis method with n-pentane or n-hexane, and 73%–98% of these products were converted into liquid hydrocarbon oils at 150°C–200°C within 15 h to 72 h. Conk *et al.* [75] improved the process and directly converted HDPE or LDPE into propylene through a series of reactions. Specifically, the method involved a small extent of desaturation of each PE chain by a dehydrogenation catalyst, followed by steady breakdown of the chains into propylene through catalytic isomerization and metathesis reactions with ethylene. The propylene yield was as high as 80%. In addition, to improve the catalytic decomposition efficiency, Jia *et al.* [76] reported the depolymerization of HDPE in various liquid-phase solvents with the Ru/C catalyst. Under optimal conditions (220°C, 2 MPa H_2_), the yields of jet-fuel and lubricant-range hydrocarbons reached 60.8% and 31.6% within 1 h, respectively. Zhang *et al.* [77] reported a new strategy that combines endothermic cleavage of the polymer C–C bonds with the exothermic alkylation reaction of the cracking products and realizes the full conversion of polyolefins into liquid iso-alkanes (C_6_ to C_10_) at temperatures below 100°C within 4 h. In addition, several researchers have noted that solvents play a very important role in direct coal liquefaction [78,79]. The physical role of solvents is to facilitate heat transfer and dissolve coal, hydrogen and liquefaction products. The chemical action of solvents is to provide activated hydrogen for coal, transfer H_2_ to coal through the hydrogen shuttle action and promote the cleavage of the C_aryl_–C_alkyl_ bond. A well-designed solvent system and suitable catalyst can realize the highly selective depolymerization of polyolefins under mild conditions. In addition, in several studies, polyolefin wastes have been dissolved in solvent oils, such as vacuum gas oil (VGO), light cycle oil (LCO) and heavy cycle oil, to realize the catalytic pyrolysis of these wastes based on existing catalytic cracking or hydrocracking processes in the oil refining field. This process changes the solid−solid reaction to liquid−solid reaction, which improves the yield of pyrolysis oil and selectivity of target products. Kohli *et al.* [80] investigated the thermal cracking of heavy crude and vacuum residues with polyolefin wastes at 420°C and 6 MPa H_2_. They demonstrated that the yield of the middle distillate fraction was maximized by minimizing the retrogressive polymerization reactions during the reaction process. Moreover, the addition of polyolefin wastes to vacuum residue feeds helped to reduce coke formation and increase metal and asphaltene removal activities. Rodríguez *et al.* [81] studied the cocracking of HDPE dissolved in VGO under conditions similar to those of the industrial FCC unit. High conversions of feedstocks and yield of naphtha and concentration of light olefins were observed in this previous study due to the synergistic effect. However, these researches only analyzed the influence of operating parameters, such as pyrolysis temperature, residence time and catalyst addition, on the product distribution during copyrolysis of plastics and solvent oils. The synergy mechanism between the two still needs further exploration.

### Gasification

The gasification of waste polyolefins is expected to increase the yield of gaseous products or syngas and reduce the yield of the main undesirable byproducts, such as tar and char. Compared with the pyrolysis technology, a remarkable advantage of gasification is the greater flexibility in jointly valorizing plastic wastes with other raw materials. The composition of gaseous products produced by plastic gasification is closely related to the gasifying agent. Air gasification has been widely used in experiments and pilot projects, its main purpose being for energy production. Steam gasification is an effective protocol for producing syngas with rich H_2_ from waste polyolefins. However, the large formation of tar during the steam gasification process remains a challenge. Currently, the strategy of combining pyrolysis with steam reforming has gained increasing attention. The gas products produced in the process are free of tar, which solves the main problem of the gasification process.

Gasification mainly involves the following steps: pyrolysis of feedstocks, cracking and reforming reactions in the gas phase, and heterogeneous char gasification. The pyrolysis process involves a series of complex endothermic chemical reactions. The low thermal conductivity and sticky nature of waste polyolefins reduce their thermal decomposition kinetics and easily lead to the formation of fused plastic agglomerates. Thus, in the gasifier design, heat transfer rates should be increased as much as possible. Meanwhile, waste polyolefins can be almost completely converted to volatiles at high heating rates. Therefore, the heterogeneous char gasification step can usually be ignored in the overall conversion. At temperatures above 800°C, the main pyrolysis mechanism of waste polyolefins mainly follows an end-chain scission, such as direct, 1,5-radical transfer and multiple step-radical transfer scissions [82]. The homogeneous gasification process involves a large number of reactions, such as steam reforming of hydrocarbons, methane reforming, dry reforming of hydrocarbons, Boudouard reaction and water–gas shift reaction. The balance of these reactions is greatly related to the type of gasifying agent, equivalence ratio or steam-to-carbon ratio (S/C) and temperature. Arena *et al.* [83] investigated the air gasification of PE in a bubbling fluidized bed using olivine as a catalyst and observed that olivine can greatly improve the efficiency of the gasification process and reduce the content of tar in gas products. The contents of H_2_, CO and CH_4_ in the gas product were 30.1–29.1, 18.4–20.9 and 3.4–1.5 vol.%, respectively. Wilk and Hofbauer [84] developed a dual fluidized bed reactor and studied the steam gasification of mixed waste polyolefins. At 850°C and S/C ratio of 2, the main gaseous product compositions were 46 vol.% H_2_, 22 vol.% CO and 16 vol.% CH_4_.

Syngas is the main product of the waste polyolefin gasification process, and it can be used to prepare olefins by Fischer-Tropsch synthesis and methanol-to-olefin technology. Olefins can be further used as feedstock to produce new plastic products, which realize the chemical recycling of polyolefin wastes.

### Other chemical recovery strategies

Several novel strategies, such as photocatalysis and electrocatalysis, for degrading plastic wastes at ambient temperature and pressure have been reported. During the photocatalytic process, photogenerated electrons (e^−^) and holes (h^+^) are generated when photon energy irradiates the catalyst. On the one hand, plastic waste can be oxidized by h^+^ [85]. On the other hand, plastic wastes can also be decomposed by hydroxyl and superoxide radicals produced from the reaction of e^−^ and h^+^ with O_2_ or H_2_O [86]. During the electrocatalytic process, oxidation and reduction reactions occur at two electrodes that are linked via an exterior electric circuit and the in-between electrolyte [87]. The externally applied potential triggers chemical reactions, which enable the conversion of plastic wastes into valuable chemicals under mild conditions. Although these methods are more energy saving and environmentally friendly and coincide with the concept of sustainable development, the reaction process requires a relatively long time period, and the conversion efficiency of plastic wastes is very low compared with that under violent conditions such as those involving pyrolysis, catalytic pyrolysis, solvolysis and gasification. In addition, these technologies are still a distance away from industrialization.

## REFINING OF PYROLYSIS OIL

The properties and composition of polyolefin pyrolysis oil have been analyzed by a number of researchers. The oil obtained in the slow pyrolysis of HDPE at 430°C has a composition similar to that of VGO, and the composition of HDPE pyrolysis oil is similar to that of LCO above 460°C [88]. Quesada *et al.* [89] analyzed the composition of pyrolysis oil of PE and PP at 500°C and reported that the composition of PE pyrolysis oil mainly included 1-alkenes and n-alkanes in the range of C_7_ to C_35_. The PP pyrolysis oil at 405°C was enriched in the naphtha range hydrocarbons with a preponderance of diesel range hydrocarbons (C_6_–C_16_: ∼50%; C_13_–C_16_: 33.04%), and the distributions of paraffinic, olefinic and naphthenic hydrocarbons were 66.55%, 25.7% and 7.58%, respectively [90]. Sinopec collected and analyzed several pyrolysis oils of polyolefin waste (Table 4). These pyrolysis oils showed a wide distillation range, covered all fractions from gasoline to heavy oil, and contained more than 30% naphthene/olefins. Based on the above properties, pyrolysis oil from polyolefin wastes is a promising feedstock for the production of liquid transportation fuels or polymer monomers (C_2_H_4_ and C_3_H_6_). The refining of pyrolysis oil is similar to that of petroleum-based feedstock. In consideration of the economy of chemical recycling of polyolefin wastes, catalytic cracking and steam cracking are usually used to refine pyrolysis oil to olefins.

**Table 4.** Composition and properties of pyrolysis oil from actual polyolefin wastes and hydrotreated pyrolysis oil.

Samples*	Oil 1#	Oil 2#	Oil 3#	Hydrotreated oil 1	Hydrotreated oil 2	Hydrotreated oil 3
Density/(20°C, kg·m^−3^)	830.9	815.5	823.6	819.2	817.2	803.4
*w*(S)/(mg·kg^−1^)	336.0	310.0	576.0	36.0	94.0	27.0
*w*(N)/(mg·kg^−1^)	629.0	421.0	2600.0	7.0	19.0	60.0
*w*(Cl)/(mg·kg^−1^)	84.0	90.0	182.0	<1.0	<1.0	<1.0
*w*(Si)/(mg·kg^−1^)	46.0	71.0	451.0	<1.0	<1.0	<1.0
*w*(O)/wt.%	0.2	0.3	0.5	<0.2	<0.2	<0.2
*w*(C)/wt.%	87.4	88.1	86.3	85.5	86.6	86.0
*w*(H)/wt.%	12.6	11.9	12.7	13.9	13.4	14.0
Hydrocarbon composition/wt.%						
Paraffins	25.4	14.3	18.6	60.4	48.0	60.2
Naphthene/olefins	52.3	39.7	63.0	23.3	20.8	19.0
Aromatics	22.3	46.0	18.4	16.2	31.2	20.8
Total	100	100	100	100	100	100

*The sample is the actual polyolefin waste, but the pyrolysis temperature and residence time are different from each other.

As shown in Table 4, high concentrations of contaminants (i.e. heteroatoms and metals) and olefins results in uncertainty during catalytic cracking and steam cracking. N, S, O, Cl, Si, and metals are the most prominent pollutants in polyolefin wastes, which can lead to serious problems, such as corrosion, fouling, increased coke formation, and downstream catalyst poisoning during the steam cracking process [91]. In addition, olefins in the steam cracker have a considerable influence on coke formation and the fouling of heat exchanger surfaces [92]. The hydrotreatment step prior to catalytic cracking and steam cracking can efficiently decrease the concentration of unsaturated hydrocarbons and remove heteroatoms and metals [93]. Our previous work (Table 4) also proved this conclusion. Therefore, hydrotreatment of pyrolysis oil prior to catalytic cracking and steam cracking is an essential step to avoid unfavorable influences. The energy consumption of the hydrogenation unit varies depending on the raw materials. The energy consumption of the diesel hydrogenation unit of Zhongke Refining and Petrochemical Company Limited is 2.77 kgEo/t, and that of the residue oil hydrogenation unit of Sinopec Maoming Petrochemical Company is 8.47 kgEo/t. However, no energy consumption data are available for the commercial operation of pyrolysis oil hydrogenation units.

The conversion of pyrolysis oil from polyolefin wastes into low-carbon olefins is one of the essential steps to realize the chemical recycling of polyolefin wastes. As the core conversion process in a refinery, catalytic cracking has an irreplaceable position in the deep processing of heavy oils to light oils and low-carbon olefins. It has the advantages of wide adaptability of raw materials, high conversion rate of heavy oil, high yield of light oil, flexible product scheme, and low operating pressure and investment [94]. Table 5 summarizes several catalytic cracking processes of polyolefin pyrolysis oil [95–99]. A competitive olefin yield can be obtained by optimizing the conditions of catalytic cracking, such as increasing space–time and temperature and selecting an appropriate catalyst. After separation, these mixed olefins can be repolymerized as feedstocks into virgin polyolefins. The energy consumption of catalytic cracking units accounts for 25%–34% of the total energy consumption of refineries. The energy consumption of the 4.2 million tons/a catalytic cracking unit of Zhongke Refining and Petrochemical Company Limited is 31.69 kgEo/t.

**Table 5.** Brief introduction of catalytic cracking of polyolefin pyrolysis oil.

Feeds	Conditions	Outputs	References
HEPE wax	Catalyst: HZSM-5 zeolite Temperature: 550^o^C	C_2_–C_3_ olefins:62.9 wt.%, ethylene: 10.6 wt.%, propylene: 35.6 wt.%, butene: 16.7 wt.%	[95]
Pyrolysis oil of polyolefin wastes	Catalyst: FCC equilibrium catalyst Temperature: 520^o^C	Ethylene:3.9 wt.%, propylene: 15.5 wt.%, gasoline: 30.5 wt.%	[96]
HEPE wax	Catalyst: FCC equilibrium catalyst Temperature: 500–560^o^C	Naphtha:23.8–31.1 wt.%, LPG fractions: 13.7–18.1 wt.%	[97]
HDPE waxes (20 wt.%) and VGO	Catalyst: FCC equilibrium catalyst Temperature: 500–560^o^C Catalyst to oil mass ratio: 3–7 g_cat_/g_feed_^−1^ Contact time: 6 s	Ethylene: 0.8–4.5 wt.%, propylene: 3.1–6.7 wt.%	[98]
PE, PP waxes and HVGO*	Catalyst: FCC equilibrium catalyst Temperature: 525^o^C Catalyst to oil mass ratio: 12 g_cat_/g_feed_^−1^ Contact time: 6 s	For PE wax, ethylene: 1.7–2.0 wt.%, propylene: 7.1–10.2 wt.%, liquid org. phase: 48.9–53.9 wt.%. For PE wax and HVGO, ethylene: 1.8–2.1 wt.%, propylene: 7.1–10.8 wt.%, liquid org. phase: 49.9–52.9 wt.% For PP wax, ethylene: 1.2–2.1 wt.%, propylene:4.3–9.5 wt.%, liquid org. phase: 50.7–67.6 wt.% For PP wax and HVGO, ethylene: 1.7–1.9 wt.%, propylene: 5.4–9.1 wt.%, liquid org. phase: 52.9–56.9 wt.%	[99]

*HVGO is the abbreviation of vacuum gas oil.

As the leading technology used in refineries, steam cracking has great potential to produce ethylene, propylene, 1,3-butadiene, etc., with the use of pyrolysis oil from polyolefin wastes [100]. In theory, the pyrolysis oil from polyolefin wastes can be directly used in steam crackers without unit modification or replacement within the refinery complex [101]. Given the large-scale operation of industrial steam crackers, co-feeding of pyrolysis oil and fossil fuel blends can reduce the contaminant levels and improve the ethylene yield [91,102]. Furthermore, as shown in the section on the pretreatment of polyolefin wastes, the systematic separation of polyolefin waste from other polymers is important to reduce the main sources of contaminants, such as nitrogen, chlorine and oxygen. As shown in Table 4, the properties of hydrogenated pyrolysis oil are similar to those of crude oil fractions. The energy consumption data of its steam cracking unit are the same as those of the current mainstream cracking unit in refineries, that is, the combustion energy consumption of olefins (ethylene + propylene) ranges from 400 kgEo/t olefins to 430 kgEo/t olefins.

Currently, numerous companies have invested considerable effort in polyolefin chemical recycling. The pyrolysis process is the main technology for most plants. Most petrochemical companies have attempted to refine pyrolysis oil to chemicals and/or fuels. Plastic Energy developed the Thermal Anaerobic Conversion technology to convert polyolefins to pyrolysis oil (850 L/t polyolefin wastes) and offered it to SABIC. Pyrolysis oil is further refined to a certified circular polymer consisting of PE and PP by SABIC [103]. In addition, Quantafuel has developed pyrolysis technology and achieved 85% pyrolysis oil yield. Its products can be used for BASF’s integrated chemical production network. With HDPE as an example, the yield (virgin plastics/waste HDPE) can reach 65% with the cooperation of Quantafuel and BASF [104]. In addition, ExxonMobil is collaborating with Plastic Energy regarding an advanced recycling plant in Notre Dame de Gravenchon, France; this plant is expected to process 25 000 metric tons of plastic waste per year by 2023, with the potential for further expansion to 33 000 metric tons of annual capacity [105]. Sinopec developed a continuous pyrolysis technology to convert polyolefin wastes to pyrolysis oil and liquefied petroleum gas with a total yield of 88 wt.%, and is building a pyrolysis unit to process 10 000 tons of polyolefin waste per year followed by refining of pyrolysis oil to virgin polyolefins in Tahe Refining and Chemical Company. Other petrochemical companies, such as Chevron and Eastman Chemical, have prepared patent portfolios in the field of refining pyrolysis oil.

Existing refining technologies have become the preferred method for refining and chemical enterprises to treat pyrolysis oil. The cooperation of plastic recycling, pretreatment, and pyrolysis plants and petrochemical enterprises will become the mainstream to realize the chemical recycling of polyolefin wastes in the future.

## LIFE CYCLE ASSESSMENT (LCA) OF POLYOLEFIN CHEMICAL RECYCLING

LCA applications have become increasingly popular to address the environmental footprints of polyolefin recycling. Given the differences in calculation models and boundary conditions, CO_2_ emissions differ substantially from each other. Rem *et al.* [106] indicated that the absolute CO_2_ emissions of polyolefin waste pretreatment were 112–119 kg CO_2_ eq./kg polyolefin waste. Jeswani *et al.* [107] analyzed the life cycle environmental impacts of chemical recycling via pyrolysis of mixed plastic waste and indicated that two-thirds of the total impact (before the credits) came from the pyrolysis process and 26% from waste collection and sorting. Schwarz *et al.* [108] indicated that waste streams with high polyolefin contents showed better environmental results with chemical recycling (gasification and pyrolysis to waxes and feedstock) than incineration with energy recovery. In addition, pyrolysis of polyolefins to monomers resulted in a CO_2_ reduction of more than 80% compared with that obtained during incineration (1.5–2.9 kg CO_2_ eq./kg polymer) [109]. Li *et al.* [96] designed several chemical recovery/recycling routes for polyolefin wastes and quantified their environmental impact through LCA. As shown in Table 6, the combustion of byproduct oil in the refinery contributed the most to the overall impacts [107]. In addition, CO_2_ emissions of the refining process, including refining of oil and polymerization of olefins, were several times higher than those of polyolefin waste pretreatment and pyrolysis. Furthermore, compared with chemical recycling of oils and polyolefins, chemical recycling of polyolefins had more advantages in the reduction of CO_2_ emissions. The results also showed that the CO_2_ emission reduction of polyolefin chemical recycling (pyrolysis→hydrotreatment of oil→steam cracking for olefins→polymerization) was more than 65.3% compared with that of incineration, which indicated good environmental performance and green characteristics.

**Table 6.** CO_2_ emissions during the whole process of chemical recycling and incineration (kg CO_2_ eq./kg polyolefin wastes).

Scheme	Pretreatment*	Pyrolysis	Refining of oil	Polymerization	Combustion of by-product oil	Total
For polyolefins	0.059	0.15	0.77	0.26	1.19	2.43
For polyolefins and oils	0.059	0.15	0.62	0.059	1.69	2.58
Incineration						6.86

*Pretreatment CO_2_ emission was estimated from the data of Jeswani *et al.* [107].

## CONCLUSION AND PERSPECTIVES

This paper reviewed the chemical recycling process of polyolefin wastes, including moderate pretreatment, pyrolysis, gasification, and refining of pyrolysis oil for olefins, and brief LCA applications were used to address the environmental footprints.

Appropriate pretreatment is necessary to provide relatively pure feedstock for the pyrolysis and refining process, and a low sorting efficiency can affect the economic and environmental performance. Thermal pyrolysis is one of the most effective chemical recovery methods for polyolefin wastes, but the wide product distribution and high impurity content limit its further application. Catalytic pyrolysis and solvolysis can improve the product distribution of pyrolysis oils to a certain extent. However, the development of cheap, efficient, and stable catalysts is still a key research direction in the future. Catalytic and steam cracking are essential processes in the production of olefins, which are the monomers of plastics from pyrolysis oil, and realization of the closed-loop recycling of polyolefin wastes. Compared with incineration, chemical recycling of polyolefin wastes shows good environmental performance and green characteristics, and it will play an essential role in the future of plastic recycling.

Facing the current chemical recycling challenge of polyolefin wastes, the following problems should be solved in the future:

1. New plastics should be developed to decrease or avoid the application of additives, and the application of composite plastics should be decreased.
2. The management of plastic wastes should aim at the use of a hierarchical approach. Targeted recycling schemes should be selected for different labeled plastic wastes.
3. The influence of impurities on pyrolysis or catalytic pyrolysis should be clarified, and antipollution catalysts should be developed. The heat, mass and momentum transfer of polyolefin fluids with a high viscosity in the reactor should be further studied.
4. More attention should be given to the development of high-value utilization technology for polyolefin wastes to avoid the destruction of polymer molecular chains. New technologies that can efficiently depolymerize polyolefin plastics into high-quality fuels with high light-component content and relatively concentrated carbon numbers under mild conditions should be developed.
5. The cost of chemical recycling of plastic wastes needs to be reduced further. For the realization of closed-loop recycling of plastic wastes, authorities should rely on existing facilities in refineries to further process pyrolysis products. This model is not only conducive to achieving carbon emission reduction in refineries but also transforming them from polluters to pollution controllers.
6. We should accelerate the low-carbon certification of pyrolysis technology and its products to promote the development of chemical recovery technology.
